# Dermoskeleton morphogenesis in zebrafish fins

**DOI:** 10.1002/dvdy.22444

**Published:** 2010-11

**Authors:** Manuel Marí-Beffa, Carmen Murciano

**Affiliations:** Department of Cell Biology, Genetics and Physiology, Faculty of Science, University of Málaga, and Biomedical Research Networking Center on Bioengineering, Biomaterials and Nanomedicine (CIBER-BBN)Málaga, Spain

**Keywords:** zebrafish, development, regeneration, fin, fin bud, rays

## Abstract

Zebrafish fins have a proximal skeleton of endochondral bones and a distal skeleton of dermal bones. Recent experimental and genetic studies are discovering mechanisms to control fin skeleton morphogenesis. Whereas the endochondral skeleton has been extensively studied, the formation of the dermal skeleton requires further revision. The shape of the dermal skeleton of the fin is generated in its distal growing margin and along a proximal growing domain. In these positions, dermoskeletal fin morphogenesis can be explained by intertissue interactions and the function of several genetic pathways. These pathways regulate patterning, size, and cell differentiation along three axes. Finally, a common genetic control of late development, regeneration, and tissue homeostasis of the fin dermoskeleton is currently being analyzed. These pathways may be responsible for the similar shape obtained after each morphogenetic process. This provides an interesting conceptual framework for future studies on this topic. *Developmental Dynamics 239:2779–2794, 2010*. © 2010 Wiley-Liss, Inc.

## INTRODUCTION TO FIN MORPHOGENESIS

**Fig. 1:**
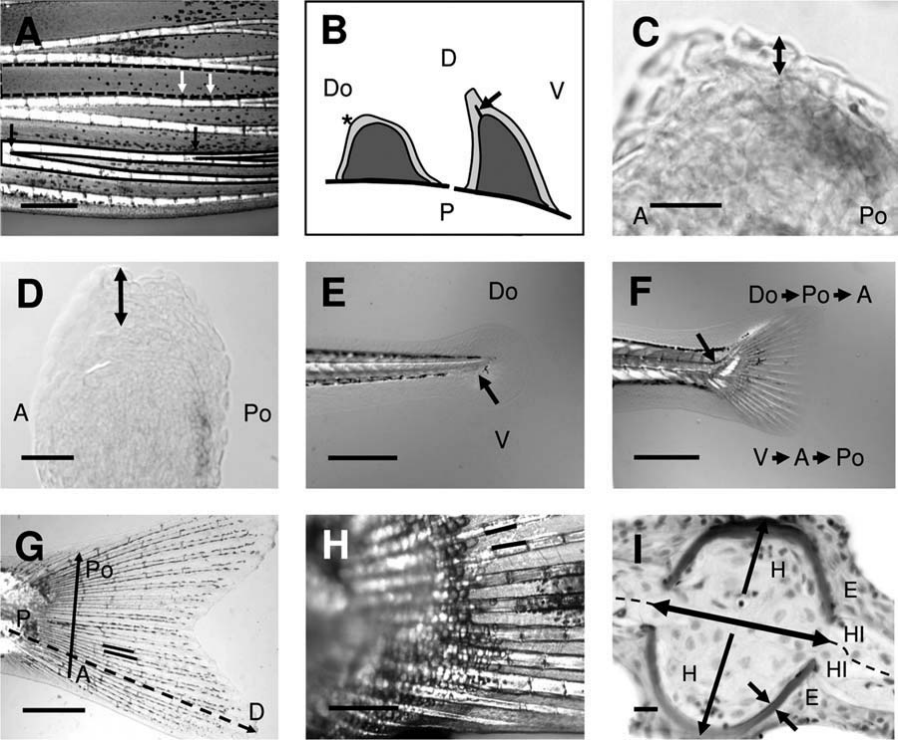
Fin development in zebrafish. **A**: Tail fin of a 29.5-mm standard length (SL, from mouth to tail fin base) individual. Continuous and discontinuous lines, respectively, delimit ray and interray regions. Black arrows show ray branching-points. White arrows show ray joints. **B**: Scheme showing formation of pectoral apical fold (arrow). Left and right, respectively, are 31- and 46-hpf fin buds. Asterisk shows apical thickening. **C, D**: In situ hybridization of *shh* mRNA in early (48 hpf, C) and late (72 hpf, D) pectoral fin buds (according to Hoffman et al., 2002). Stained regions at Po are ZPA. Double-arrows label AER (C) and fin fold (D). **E–H**: Tail fins of a 4.5- (E), 5.5- (F), 8.5- (G) and 26.5-mm (H) SL zebrafish. Arrows show ACFP (E) and notochord dorsal bending (F). Small arrows indicate axis renaming (F). Discontinuous and continuous arrows are morphological proximodistal and anteroposterior axes (G). Length and distance between double-line are inter-joint distance and inter-ray width (G,H). **I**: Ray cross section. H, HI, and E, respectively, show hemi-ray, hemi-inter-ray, and epidermis. Contralateral divergent arrows show ray thickness. Double arrow shows ray width. Opposed arrows show lepidotrichia thickness. Do, V, Po, A, P, and D, dorsal, ventral, posterior, anterior, proximal, and distal, respectively. A, C–H: Nomarski optics. Scale bar = 10 (I), 40 (C), 80 (D), and 500 μm (A, E–H).

Actinopterygian fishes show paired (pectoral and pelvic) and median (dorsal, anal and caudal) fins. The fin skeleton consists of a proximal endochondral skeleton and a distal dermal skeleton. The bones of proximal skeleton are characteristic of each fin (Grandel and Schulte-Merker, 1998; Bird and Mabee, 2003). The fin dermal skeleton of actinopterygian fishes consists of spines and/or soft-rays connected by interspines or interrays. Each soft-ray (referred to as ray) is formed of two apposed hemirays that are contralaterally symmetrical (left and right in median fins, dorsal and ventral in paired fins). Each hemiray includes a dermal bone called lepidotrichium (e.g., Becerra et al., 1983 and references within) or lepidotrich (Grandel and Schulte-Merker, 1998). The lepidotrichium of each hemiray, or half ray (Grandel and Schulte-Merker, 1998), is segmented by joints and branched several times along the proximodistal axis (Fig. 1A, e.g., Becerra et al., 1983). The exact pattern also varies along the anteroposterior fin axis. At distal ray positions, actinotrichia also develop. These actinotrichia are colagenous macrofibrils organized into two contralateral palisades. These skeletal elements are immersed in a loose connective tissue with blood vessels and nerves, and surrounded by a stratified epidermis (Becerra et al., 1983). Maintenance of the distal dermal skeleton has been shown to depend on adult tissue renewal, in a process named homeostatic regeneration (Wills et al., 2008).

Paired and median fins have different embryological origins but share similar developmental mechanisms (Freitas et al., 2006). Two main organizers have been proposed to control the development of both fin types (e.g., Neumann et al., 1999; Grandel et al., 2000; Fischer et al., 2003; Mercader et al., 2006; Nomura et al., 2006; see below). These fin organizers of fishes are probably conserved since basal gnatostomes (see Freitas et al., 2006) and might reflect the ancestral state of vertebrate appendage organizers (e.g., Sordino et al., 1995; Freitas et al., 2006; Dahn et al., 2007; Hadzhiev et al., 2007).

A lateral plate mesoderm induction initiates pectoral fin development in zebrafish. Two organizers are formed during the process: the apical ectodermal ridge (AER), and the zone of polarizing activity (ZPA). The apical ectodermal ridge (Fig. 1B,C, e.g., Fischer et al., 2003; Mercader et al., 2006; Nomura et al., 2006), also named AER-like ridge (Hinchliffe, 2002), is homologous to tetrapod limb AER (e.g., Neumann et al., 1999; Fischer et al., 2003; Mercader, 2007). AER is an apical thickening that shows a very small fold at its basal stratum (Fig. 1B). AER distally covers the fin bud along the dorsoventral interface. In zebrafish fin bud, mesenchymal cells are proposed to derive from lateral plate and proliferate to form the bud (Ahn et al., 2002; Grandel and Schulte-Merker, 1998; for killifish see Wood and Thorogood, 1984). A fate map in *Salmo trutta fario* showed that this mesenchyme secondarily gives rise to the fin endoskeleton (Bouvet, 1971). During development, the AER further folds giving rise to the fin fold (Fig. 1B). The fin fold, with underlying actinotrichia (Fig. 1D; e.g., Sengel and Bouvet, 1968; Bouvet, 1971; Grandel and Schulte-Merker, 1998; Neumann et al., 1999) and infiltrating mesenchyme (Wood and Thorogood, 1984), gives rise to the distal dermal skeleton (Bouvet, 1971). Fin fold mesenchyme has been proposed to derive from neural crest (Thorogood, 1991). Both AER and fin fold have been considered a unique transient distal organizer, the apical fold (AF, see Grandel and Schulte-Merker, 1998; Neumann et al., 1999) or the apical ectodermal fold (AEF, Freitas et al., 2006). Finally, the ZPA (Fig. 1C,D) is a second organizer in the posterior mesenchyme (e.g., Neumann et al., 1999; Grandel et al., 2000), previously observed in tetrapods (see Johnson and Tabin, 1997; see below).

In addition, median fins exist at either dorsal or ventral midlines. The adult tail fin can also be understood as a ventral fin, grown after a dorsal flexion of caudal notochord (Fig. 1E,F; e.g., Hadzhiev et al., 2007). Thus, the dorsoventral axis of the adult tail fin is indeed determined along an anteroposterior axis (Fig. 1F). Dorsal in the adult tail fin should be considered posterior, and ventral positions anterior. Structural and molecular similarities with AEF have been described in median fins (see Akimenko et al., 1994, 1995; Yonei-Tamura et al., 1999; Monnot et al., 1999; Abe et al., 2007). In zebrafish, only the tail fin fold mesenchyme has been proposed to derive from neural crest cells (Smith et al., 1994). However, histological studies of endoskeleton and dermoskeleton of chondrichthyan median fins have, respectively, suggested a sclerotome (Freitas et al., 2006) and neural crest (Freitas et al., 2006) origin of infiltrating cells. Finally, the molecular regulation of an adult caudal fin primordium (ACFP; Fig. 1E) during fin fold formation has been analyzed showing important similarities with the ZPA (Hadzhiev et al., 2007). The position of the ACFP organizer also suggests a new change in caudal fin axes (Fig. 1F). According to axis polarity of other fins (Neumann et al., 1999; Grandel et al., 2000; Freitas et al., 2006), dorsal should be now considered anterior, and ventral (near the organizer) posterior (Fig. 1F).

During the early evolution of tetrapod limbs, the fin fold and dermal skeleton of paired fins disappeared and the endoskeleton grew in size (see Holmgren, 1933). Median fins evolved before paired fins (Coates, 1994; Janvier, 1996), and the developmental control of both appendages is conserved in tetrapod appendages (Freitas et al., 2006; Dahn et al., 2007). This suggests that paired fins, and derived tetrapod limbs, have “co-opted” their genetic mechanism from median fins (e.g., Mabee et al., 2002; Freitas et al., 2006).

During the early development of the fin dermoskeleton, rays are formed separated by well-defined interrays (see Grandel and Schulte-Merker, 1998). Resembling the caudal growth of the body, the proximodistal axis appears by sequential addition of newly formed distal tissue. During the latter process, intercalary growth is not observed (Fig. 1G,H). However, the anteroposterior axis appears by intercalary growth of ray and interray widening (Fig. 1G,H). Moreover, growth along a third contralateral axis (left-right in median fins, and dorsoventral in paired fins) also occurs. During development, the contralateral thickness of the ray grows in size, thus increasing the distance between the apposed hemirays (Fig. 1I).

Macroscopically, the shape of the fin dermal skeleton can be described by a reduced number of characters. Ray length (Iovine and Johnson, 2000) and ray or interray width are characters that grow with body size or standard length (SL, see Fig. 1F–H). However, both ray branching position (see below) and inter-joint distances do not significantly grow once they are formed (see Fig. 1A, G, H). Moreover, anteroposterior polarity (e.g., Neumann et al., 1999; Hoffman et al., 2002, see below) and proximodistal polarity and direction (see Nabrit, 1929, for *Fundulus heteroclitus* data) are also characters revealed by experiments. Previous studies further suggest that most of the above-mentioned morphometric characters can be independently perturbed (Nabrit, 1929; Murciano et al., 2007).

Several very old observations state that ablation of any adult fin dermoskeleton (e.g., in *Fundulus heteroclitus*; Morrill, 1906) is followed by complete regeneration (Broussonet, 1786; Morgan, 1902; Morrill, 1906). After a fin cut in adult zebrafish, a blastema is formed by cell dedifferentiation, migration, and proliferation (Poleo et al., 2001; Santos-Ruiz et al., 2002) and it shows characteristic domains of gene expression. *eve1* and *evx2* genes are initially expressed at distal regions of the early blastema, showing variations along the anteroposterior fin axis (Brulfert et al., 1998). Moreover, *shh* pathway genes are expressed in both contralateral sides of the proximal regions of the early blastema (Laforest et al., 1998). In these proximal positions, contralateral lepidotrichia is being synthesized by scleroblasts (Becerra et al., 1996). This suggests that the anteroposterior and contralateral (left-right or dorsoventral) pattern is established during blastema formation. The outgrowth of the fin blastema is a secondary process that regenerates absent tissues by distal addition along the proximodistal axis (Broussonet, 1786). During both development and regeneration, lepidotrichia thickness also grows gradually (Fig. 1I).

Any ray (Goss and Stagg, 1957, Murciano et al., 2002) or hemiray (Goss and Stagg, 1957, Murciano et al., 2007) can regenerate anywhere in the fin after grafting. The ray is regenerated by two contralateral populations of proliferating cells, the hemiblastemas. In isolation, a hemiblastema usually regenerates a single hemiray (Murciano et al., 2007). Several domains have been found in the hemiblastema. A distal population of hemiblastemal cells stays proliferative quiescent and it expresses specific genes. In proximal positions, blastemal cells are organized in an intermediate population of proliferating cells and a proximalmost population of differentiating cells (Santamaría et al., 1996; Nechiporuk and Keating, 2002; Murciano et al., 2007). Beside this, gene expression studies further suggest several other blastema “compartments” (see Nechiporuk and Keating, 2002; Poss et al., 2002; Lee et al., 2009; Yoshinari et al., 2009; Brown et al., 2009). In any case, DiI labeling of dedifferentiating cells in the stump suggests the absence of cell lineage restrictions in the epidermis (Poleo et al., 2001) or the ray-interray mesenchyme (Murciano et al., 2007) during early fin regeneration.

During embryogenesis, rays and interrays are narrow compared to adult ones. Distal growth involves generation of increasingly wider/thicker rays/interrays and ray branching. In order to generate a plain functional structure (Alben et al., 2007), proximal development continuously widens and thickens rays and interrays in all proximodistal positions (Fig. 1G–I). During regeneration, a similar process of distal patterning and proximal widening and thickening has also been described (Murciano et al., 2007). In conclusion, ray and interray pattern and form depend on two processes: patterning/size/differentiation at the distal growing margin (DGM), and widening/thickening in a proximal growing domain (PGD).

## THE DISTAL GROWING MARGIN

**Fig. 2:**
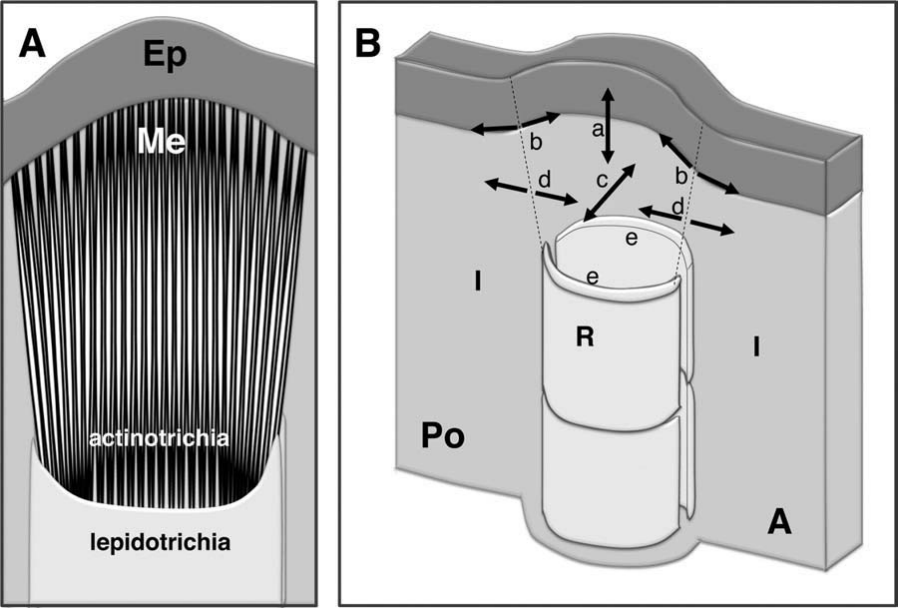
Distal growing margin hypothesis. **A**: Scheme of the microscopic anatomy of the distal growing margin (DGM). Ep, distal epidermis; Me, distal mesenchyme. The fiber bundle is the distal actinotrichia. Lepidotrichia are contralateral hemirays. **B**: Inter-tissue interactions may occur as a 3D orthogonal system at the DGM of each ray and interray (pinnamere) during development and regeneration. Po, posterior; A, anterior; R, ray; I, inter-ray. a–d arrows are the candidate intertissue/genetic interactions discussed in text. a occurs at the distal organizer. b and d occur across the ray-interray boundary organizer. b and c interactions show uncertainties on the tissue/s in which they are exerted (epidermis and/or mesenchyme). e has not been consistently related to any intertissue/genetic interaction, a genes are *fgfs*, *fgfr1*, *rarγ*; or *wnt5b*. b genes may be *rarγ*, *eve1*, or *evx2*. c genes may be Shh pathway, *cx43*, or the gene mutated in another long fin. d genes are those of ActβA or Shh pathway. e genes may be *evx1*, *hoxa13b*, or *cx43*.

**Fig. 3:**
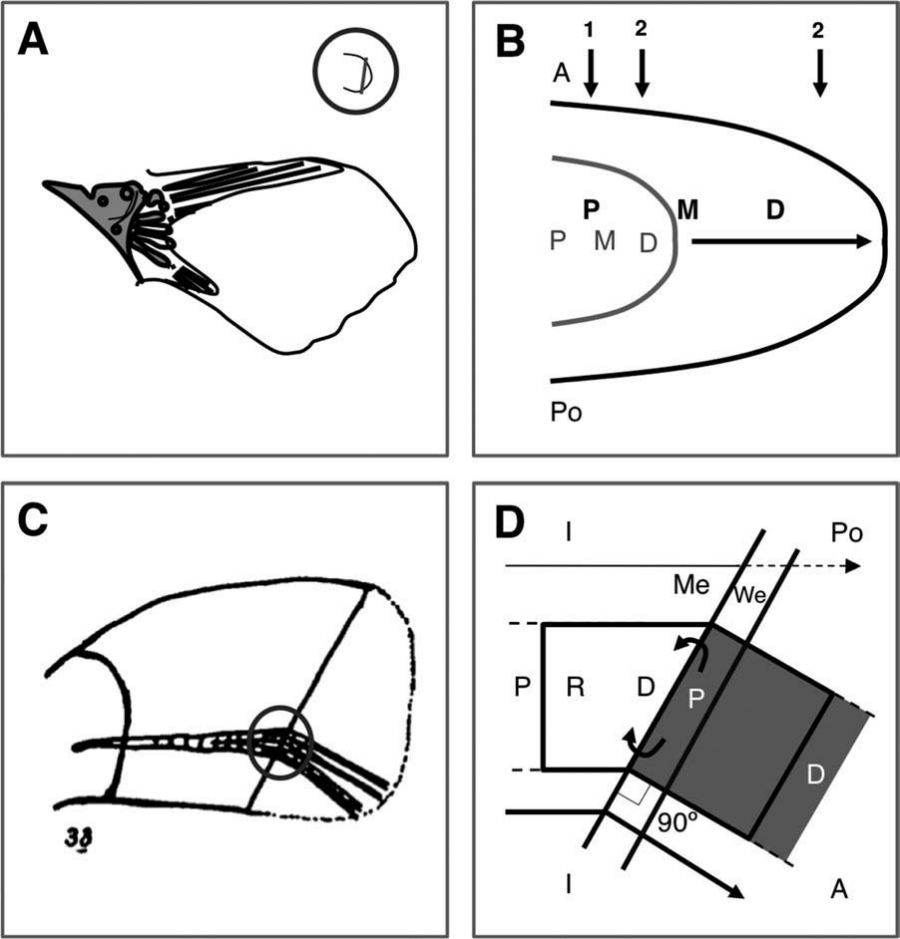
Experiments on a specification map and outgrowth direction. **A**: Distal ablation of early pectoral fin bud and grown morphology in Salmo trutta fario (according to Bouvet, 1971). Grey, present; white, absent. **B**: Scheme showing proximal intercalary growth (distance increase between P, M [Medial] and D fates) and distalization (new fates distal to D) during early fin development. 1 and 2 are cut positions explained in text. Grey, an earlier stage. **C**: Perpendicular regenerate following oblique cut in Fundulus tail fin. C is reproduced from Nabrit (1929). **D**: Scheme of potential wound epidermal (We)-mesenchymal (Me) interactions that generate the results shown in C. Curved arrows, We-Me interactions; thin arrow, original proximodistal ray polarity and direction (discontinuous in regenerated regions shown in grey); thick arrow, grown proximodistal polarity and direction; 90° and small right angle, outgrowth angle with respect to the cut plane. Position and axis symbols are as in Figure 1. Grey circles in A and C, respectively, show the fin regions drawn in B and D.

**Fig. 4:**
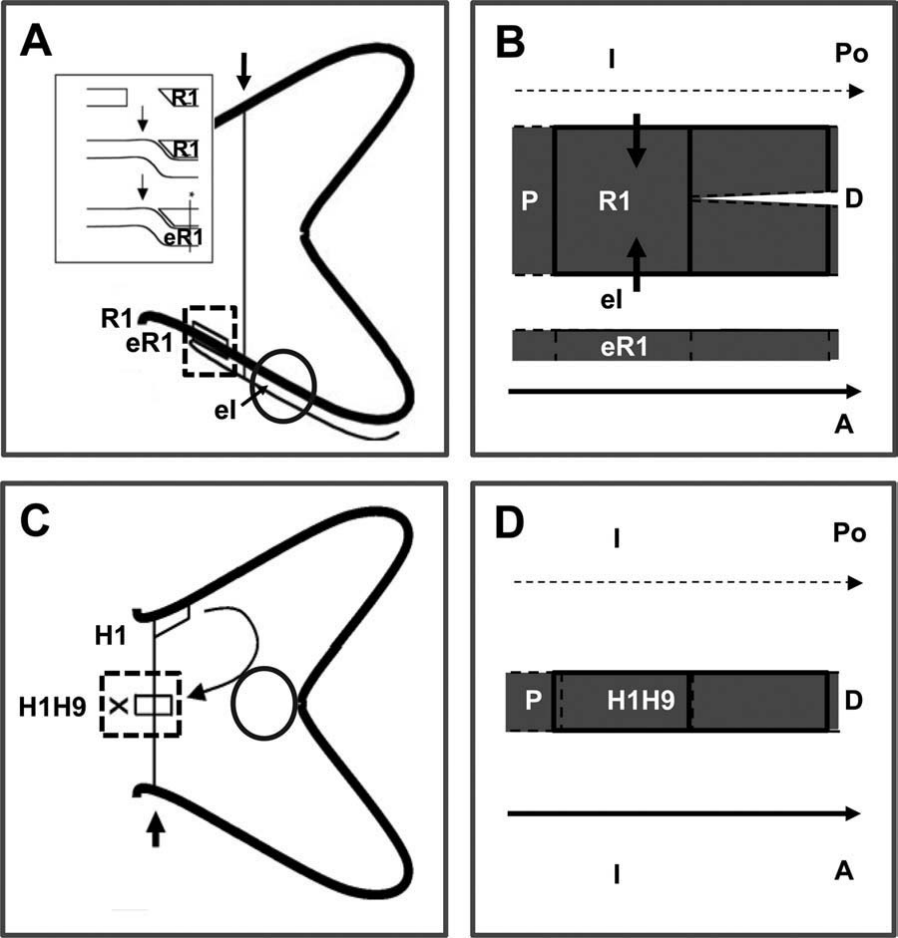
Experiments on local morphogenesis control. **A**: Proximal hole with distal oblique cut in R1 (discontinuous rectangle and inset) and regenerated ectopic R1 (eR1). eR1 outgrows outside the fin and is joined to R1 by an ectopic interray (eI). The larger arrow is a subsequent distal, transversal cut. **B**: Following the operation in A, the experimental ray1 regenerate (Ray 1) may show branching. Ray branching may depend on ectopic interactions from neighboring tissues (small, thick arrows). Ray/interray symbols are as in A. **C**: A recombinant H1H9 ray (discontinuous rectangle) is obtained substituting by grafting (curved arrow) a hemiray 9 (H9) by a hemiray fragment (small rectangle) from ray 1 (H1). H1H9 regenerate is obtained following fin cut (small thick arrow). **D**: Registered joint positioning at distal H1H9 regenerate. Continuous and discontinuous transversal lines show joints in contralateral hemirays. A, C: Reproduced from Murciano et al. (2002, 2007, respectively) with permission of the publisher. A and C have been clockwise rotated 90°. Definitions are as in Figure 3.

A number of articles dealing with the molecular control of the morphogenesis of the fin dermoskeleton have been recently published. Reviewed data are providing support to the notion of a common control of fin morphogenesis during fin development, regeneration, and tissue renewal at the DGM. In principle, actinotrichia are a structural element of this DGM in rays. Actinotrichia appear at the distal growing margin since the fin fold stage until adulthood, and during fin regeneration (Fig. 2A; see Géraudie and Landis, 1982). Also, a distal covering epidermis and underlying proliferating mesenchyme (Fig. 2A) with blood vessels are present during these three processes. Moreover, the intrinsic connectivity of fin morphogenesis at the DGM during development, regeneration (Fig. 2B), and tissue renewal is being studied. Intertissue interactions and gene functions are being discovered by grafting, mutant/transgenic studies, morpholino/plasmid injections, and/or pharmacological experiments (more than 90 reports have been reviewed). Several gene functions have been shown to equally control either fin regeneration and tissue homeostasis, or fin development and regeneration. Moreover, ray growth rate (Morgan, 1902), proximodistal patterning (Nabrit, 1929; Bouvet, 1971; Fig. 3); ray, hemirray and interray morphogenesis (Nabrit, 1929; Goss and Stagg, 1957; Murciano et al., 2002, 2007; Fig. 4), and the expression of some developmental genes (Murciano et al., 2002; see Chablais and Jaźwińska, 2010) locally depend on intertissue interactions (Fig. 2B; see Nabrit, 1929). Those intertissue interactions take place between rays, interrays, and the surrounding epidermis. In the current article, the DGM will be considered as the composition of interactive controlling units in each distal ray and both neighboring interrays during the three morphogenetic processes. We have preliminarily named *pinnamere* (from “unit of fins” in Latin) DGM to each of these interacting units. These intertissue interactions depend on both a distal and a ray-interray boundary organizer and regulate *pinnamere* morphology. Fin development, regeneration, and tissue renewal may share a regulative commonality, which is also used here to explain the similar resulting fin forms.

## MORPHOGENESIS AT THE DISTAL GROWING MARGIN

**Fig. 5:**
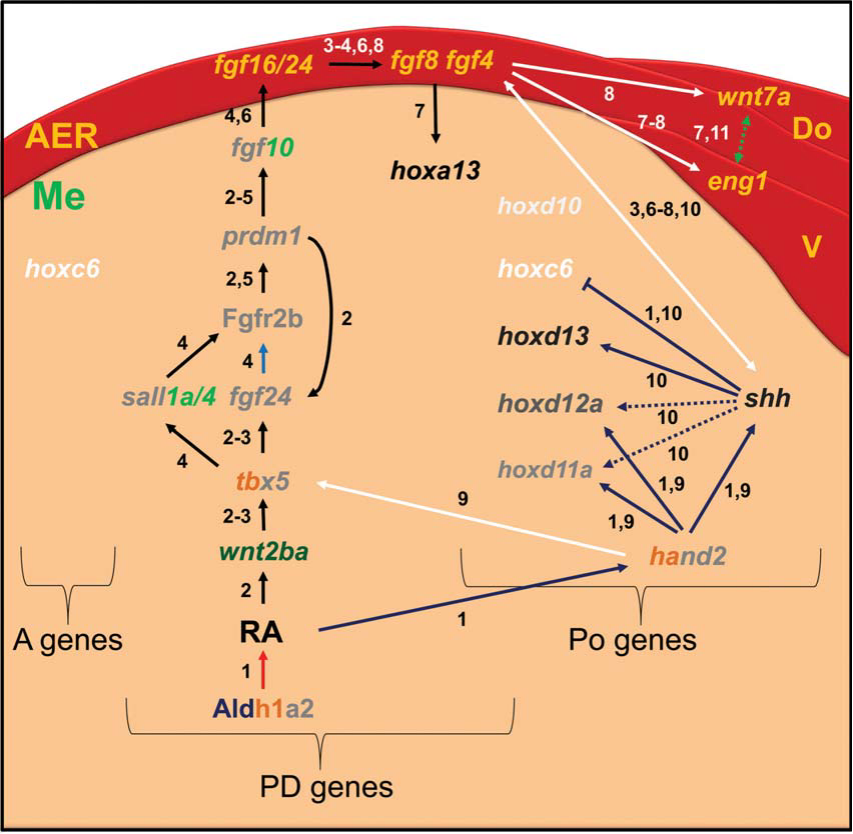
Interactions among axis genes in the early pectoral fin bud. Black, blue, and green arrows are interactions between proximodistal, anteroposterior, and dorsoventral (contralateral) regulatory genes, respectively. Aldh1a2 initiates these interactions from the somites. Suggested interactions are subsequent gene transcription activation. Light blue arrow, ligand-receptor interaction; red arrow, enzyme-product relationship. The arrows from *hand2* and *shh* genes show initiation control. Dotted arrows from these genes show maintenance control. Broken arrows, repression; white arrows, interactions among proximodistal, anteroposterior, and dorsoventral genes; other discontinuous arrow, uncertainty. Orange genes are expressed in the AER. Gene colors and definitions are as in Figures 1, 2, and 7. Several-colored genes show various expression domains at different stages. 1, Gibert et al. (2006); 2, Mercader et al. (2006); 3, Fischer et al. (2003); 4, Harvey and Logan (2006); 5, Lee and Roy (2006); 6, Nomura et al. (2006); 7, Grandel et al. (2000); 8, Norton et al. (2005); 9, Yelon et al. (2000); 10, Neumann et al. (1999); 11, Hatta et al. (1991).

Our research has reviewed the genetic control of initiation and morphogenesis of fin dermoskeleton. In order to better understand its embryological origin, we will also compare early patterning of fin and limb endoskeleton (Fig. 5; see Freitas et al., 2006; Dahn et al., 2007). Based on this evidence, a positional model will be proposed to explain fin dermoskeleton morphogenesis under this controlling network. Our proposed model integrates experimental data on intertissue interactions and genetic analysis.

**Fig. 6:**
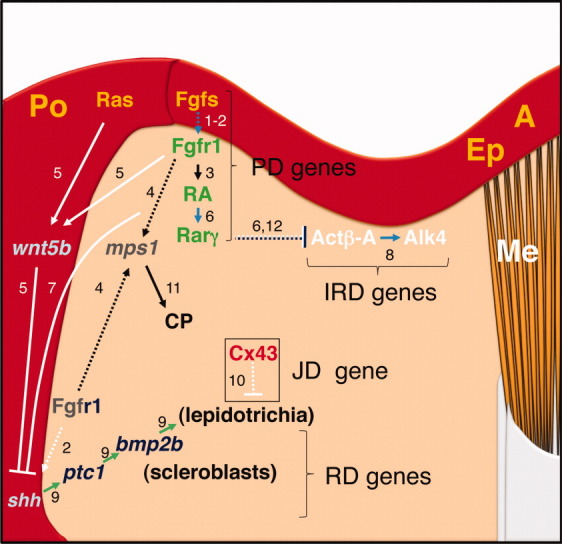
Proposed interactions among proximodistal and ray, interray and joint differentiation genes in the fin blastema. The scheme shows a lateral view of two ray blastema and an intermediate interray. To the left, the ray DGM skeleton is absent. Fgfs (e.g., Wfgf or Fgf20a; see 1,2, 4; Smith et al., 2008; Whitehead et al., 2005) may be regulated by Wnt10a/Wnt5b (Stoick-Cooper et al., 2007). CP, cell proliferation at blastema mesenchyme (Me). Fgfr1/ERK and Wnt signaling pathways regulate *raldh2*-dependent retinoic acid-synthesis at distalmost blastema (3). Gene colors are as in Figure 7E and H. PD, RD, IRD, and JD genes, respectively, regulate the proximodistal axis, ray, interray, and joint differentiation (small box) during blastema formation. Symbols and colors are as in Figure 5. Blue/white broken arrows, PD-IRD interaction along the anteroposterior axis. 1, Lee et al. (2005); 2, Poss et al. (2000); 3, Mathew et al. (2009); 4, Yin and Poss (2008); 5, Lee et al. (2009); 6, White et al. (1994); Murciano et al. (personal communication); 7, Laforest et al. (1998); 8, Jaźwińska et al. (2007); 9, Quint et al. (2002); 10, Sims et al. (2009); 11, Poss et al. (2002); 12, Brulfert et al. (1998).

**Fig. 7:**
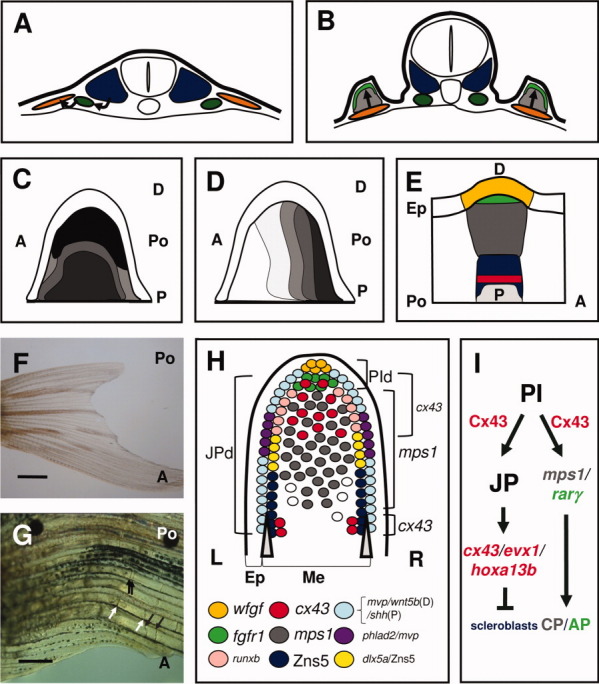
Gene expression domains during fin morphogenesis. **A,B**: Schemes of transversal sections of zebrafish embryos at 18 hpf (A, according to Mercader, 2007) and 40 hpf (B, according to Harvey and Logan, 2006). Blue, green, and dark orange, respectively, are somites, intermediate, and lateral plate mesoderms. Arrows, suggested genetic interactions. C,D: Expression domains of hox genes in Figure 5 (similar grey hue). **C**: Darker to lighter grey, respectively, illustrates *hoxa9/hoxa13*, *hoxa9/hoxa10/hoxa11*, *hoxa9/hoxa10* and *hoxa9* domains. **D:** Except for *hoxc6*, expression domains of 5′ hoxd genes are posteriorly overlapped. **E**: Gene expression domains in Figure 6 (similar color). **F**: Adult tail fin of a *long fin* mutant. **G**: Proximoanterior region of an *alf^ty86d^* tail fin. Arrows in same color show out-of-register joints. **H**: Gene expression domains (brackets and circles in same color) is ray blastema (according to Poss et al., 2000, 2002; Yoshinari et al., 2009; Brown et al., 2009). Overlapping regions, co-expression domains; PId and JPd, potential positional identity (PI) and joint positioning (JP) domains; triangles, lepidotrichia; R, right; L, left. Zns5 is an antibody. **I**: PI independently regulate (arrows) size (right) and joint positioning (left) genes. CP/AP is a balance between cell proliferation and apoptosis. Broken arrow, repression. Lettering as in Figures 1, 3–6. Scale bar = 500 (G) and 2,000 μm (F).

Genes controlling fin dermoskeleton morphogenesis have been classified in patterning, size, and tissue/cell differentiation genes. Some genes may regulate ray/interray (e.g., retinoic acid pathway) or lepidotrichia/actinotrichia (e.g., genes regulating Sonic hedgehog pathway) pattern. Size genes may control fin dermoskeleton length, anteroposterior size, or joint positioning in distal fin dermoskeleton (e.g. *fgfr1*-pathway, retinoic acid, or *connexin 43*). But also, tissue/cell differentiation genes regulate ray, interray (*shh* and *actβa/alk4*-pathways), and joint (*alf*^*ty86d*^ or *fgr1*) differentiation. These genetic functions at the DGM (see Figs. 6, 7) may give rise to the final form of the fin dermoskeleton. Specific differences among the molecular control of each fin have also been described (e.g., Fischer et al., 2003; Sumanas et al., 2002).

In general, fin development, tissue renewal, and regeneration may not be comparable (Marí-Beffa et al., 2007). A previous article shows that the caudal fin fold and the blastema only share 35 out of 250 transcripts (Yoshinari et al., 2009). Thus, gene expression in early fin fold development and regeneration are not comparable. However, *shh, ptc1, apoE, evx1, angptl2* (Laforest et al., 1998, Monnot et al., 1999; Borday et al., 2001; Kubota et al., 2005), and with slight differences *hoxa11b* or *hoxa13b* (Géraudie and Birraux, 2003) show similar expression domains during late fin fold development and adult regeneration. Furthermore, *lof*^*dt2*^ (Géraudie et al., 1993), *connexin43/sof* (*cx43*; e.g., Iovine and Johnson, 2000; Hoptak-Solga et al., 2007; Sims et al., 2009) and *alf*^*ty86d*^ (Murciano et al., 2007) mutants show similar phenotypes after these two processes. Finally, Fgfr1 (Lee et al., 2005) Fgf20a (Whitehead et al., 2005), kinase Mps1 (Poss et al., 2002), *mkp3* and *msxb* have been proposed to have similar functions (Wills et al., 2008, see below) and/or expression domains (see fig. S3 in Wills et al., 2008) during fin dermoskeleton regeneration and adult homeostasis. In conclusion, these molecular data support the hypothesis of a comparable DGM during late (but not early) fin fold development, adulthood, and regeneration of the dermal skeleton of the fin.

In this report, we have also considered these potential genetic and intertissue interactions to be organized in an orthogonal system of three axes (Figs. 5,6): (1) proximodistal, (2) anteroposterior, and (3) contralateral (dorsoventral for paired fins, left-right for median fins) axes (Fig. 2B).

### Regulative Interactions Along the Proximodistal (PD) Axis

Throughout the development of the *Salmo trutta fario*, distal regions of pectoral the fin bud and/or fin fold were cut (Bouvet, 1971, Fig. 3A). Fishes with experimental fin buds were grown and adult fins were studied (Fig. 3A). In most cases, absent skeletal elements involved both dermal and endochondral fin bones in a proximodistal series. After transversal cuts at constant absolute positions along the proximodistal axis, the resulting fins (Bouvet, 1971; 1 in Fig. 3B) lead us to a first conclusion. The earlier these cuts are carried out, the lesser skeletal eliminations are finally obtained (Fig. 3c5 vs. Fig. 3a4 or 3b5 in Bouvet, 1971). This can be explained by proximodistal intercalary growth between specified cells in the fin bud (Figure 3B). However, when fin cuts were done at a specific percentage position (e.g., distal at about 80% of the total proximodistal size; 2 in Fig. 3B), the earlier these cuts, the larger skeletal eliminations were obtained (Fig. 3c5 vs. Fig. 3d3 in Bouvet, 1971). This may be explained by differential intercalary growth and/or distal acquisition of new cell fates (distalization, Fig. 3B). As stated above, intercalary growth may exclusively affect endoskeleton development or early dermal skeleton initiation. Distalization, however, might involve both endoskeleton and dermal skeleton growth (Bouvet, 1971; see Grandel and Schulte-Merker, 1998).

A genetic mechanism controls the initiation of the pectoral fin and the AER activity during zebrafish embryogenesis (e.g., Garrity et al., 2002; Norton et al., 2005; Mercader et al., 2006; Mercader, 2007). In the current essay, we show a very simplified version of the published patterning mechanism. Retinoic acid (RA) is synthesized in the somites (e.g., Gibert et al., 2006). RA activates *wnt2ba* transcription in the intermediate mesoderm, Wnt2ba might activate *tbx5* transcription in lateral plate mesoderm, and Tbx5 initiates the formation of the pectoral fin (Figs. 5, 7A–D; Ng et al., 2002; Mercader et al., 2006; for counterview see Fischer et al., 2003; Gibert et al., 2006). Through a transcription cascade, Tbx5 ultimately regulates ectoderm expression of *fgf4* and *fgf8* (Fig. 5). This pathway has been suggested to possibly regulate a partially collinear pattern of 5′*hoxa* genes (Figs. 5, 7C; see Sordino et al., 1995; Grandel et al., 2000).

This transcriptional cascade (Fig. 5 and references within) has been shown to regulate both proximal (scapulocoracoid) and distal endoskeletal fates (e.g., endoskeletal disc). Distalization might be dependent on both cascade activity (Norton et al., 2005; Mercader et al., 2006) and AER signaling (Neumann et al., 1999; Grandel et al., 2000). Moreover, the reduction of *sall1a/4* function (Harvey and Logan, 2006) shows size reduction in specific bones intercalated along the proximodistal axis. These data are suggestive of a hierarchical quantitative control of distalization and intercalary growth. In these terms, molecular experiments provide an initial explanation to classical experiments by Bouvet (Fig. 3A,B).

The fin AER-independent “pre-pattern” and the intercalated regulation of bone morphogenesis cannot be explained by the classical model of progress zone (Saunders, 2002). An alternative hypothesis, such as the “early specification” model (Dudley et al., 2002), has been proposed to explain similar regulatory behavior in the tetrapod limb bud. Previous reports (e.g., Mercader et al., 1999; Mariani et al., 2008; see Mercader, 2007) have extensively discussed this double patterning dependence on lateral mesoderm and distal AER signals in both tetrapod limb and fin buds (see also Saunders, 1948; Niswander et al., 1993; Logan, 2003; Mariani et al., 2008). This early patterning at fin bud could be inherited by the dermal component of the fin.

Subsequent transition from the AER to the pectoral fin fold has been initially studied (Webb et al., 2007). However, the specific relationship between the AEF and early morphogenesis of the fin dermoskeleton has rarely been studied (e.g., Draper et al., 2003). Draper and colleagues argue that the embryonic injection of *fgf24*-morpholino has disclosed a *fgf24* inherited requirement in the formation of the dermal skeleton of the pectoral fin during later development (Draper et al., 2003). This interesting aspect is almost unique in the discussed scientific literature.

During late development and regeneration, a genetic control (Figs. 6, 7E) may also regulate pattern and size along the PD axis. Mutations in *long fin* (*lof*^*dt2*^) locus lead to an increase in proximodistal size (Fig. 7F; Géraudie et al., 1995; Iovine and Johnson, 2000). *rapunzel* (*rpz*) mutation shows a similar phenotype only during development (see Green et al., 2009). A molecular study on these genes suggests that this occurs by gene over-expression and up-regulation of skeletal genes (Green et al., 2009). Such studies on gene over-expression support the idea of a quantitative control of size along the proximodistal axis by these two genes.

During the fin regeneration process, epidermis-mesenchyme interactions have also been proposed to regulate proximodistal, growth rate, patterning, and size (Marí-Beffa et al., 1996; Laforest et al., 1998; Murciano et al., 2002; Chablais and Jaźwińska, 2010). When oblique cuts were done in a tail fin of *Fundulus*, the direction of the regenerated rays occurred at a 90° angle to the cut plane (Fig. 3C,D; Nabrit, 1929). After ray grafting, the distal mesenchyme of the graft was shown to ectopically induce *msxa* and *msxd* gene expression on the covering epidermis (Murciano et al., 2002). Other epidermal characters, epidermis-mesenchyme cross-interaction, and fin outgrowth have also been shown to be mediated by Igf signaling from distal mesenchyme (Chablais and Jaźwińska, 2010). During this process, epidermis-mesenchyme interactions control the initial outgrowth direction (Fig. 3D) and gene expression.

Several experiments on *fgfr1* activity have been carried out by Kenneth D. Poss' research group using a useful heat-inducible transgenic of a dominant-negative form (*hsp70:dn-fgfr1*; Lee et al., 2005; Wills et al., 2008; Lee et al., 2009). In one experiment, these researchers inactivated adult Fgfr1 for 30 days, cut the tail fin, and then restored the fish to a permissive temperature for 15 days of regeneration (Lee et al., 2005). In many instances, complete regeneration was observed. This led the authors to conclude that Fgfr1 activity does not control positional memory previous to regeneration (Lee et al., 2005).

The concrete genetic network transducing this “pre-pattern” of positional identity has been analyzed. In the regeneration process, the expression of *msxb* gene is higher in proximal blastemas and gradually lower in distal blastemas along the proximodistal axis (Akimenko et al., 1995). *msxb* knock-down by morpholino injection in ray blastema suggests that *msxb* is involved in growth rate control (Thummel et al., 2006). During the fin regeneration of the zebrafish (Poss et al., 2000; Lee et al., 2005, Yin and Poss, 2008) and of two *Xiphophorus* species (Offen et al., 2008), *fgfr1* is homogeneously expressed along the proximodistal axis and it may regulate growth rate and *msx* gene expressions. In zebrafish, Fgfr1 also regulates the proximodistal gradient expression of *mkp3, sef*, or *spry4* genes (Lee et al., 2005). Fgfr1 control of growth rate is exerted by *miR-133* mediation (Yin and Poss, 2008). This mediation regulates cell proliferation by *msp1* transcription (Fig. 6; Poss et al., 2002). Retinoic acid synthesis has also been proposed to be regulated by Fgfr1 (Mathew et al., 2009) and to control proximodistal patterning (White et al., 1994). Moreover, upstream Wnts and Fgfs in the distal epidermis and mesenchyme have been shown to instruct Fgfr1 to regulate the formation and outgrowth of fin blastema (Stoick-Cooper et al., 2007; Smith et al., 2008; Lee et al, 2009). In principle, a distal organizer-dependent gradient of “positional identity” might quantitatively control growth rate (Lee et al., 2005) and patterning along the proximodistal axis. This is a first explanation of epidermis-mesenchyme interactions (Fig. 3C,D) in molecular terms. Fgfs (Lee et al., 2005), two *hox* genes, *hoxc13a* and *hoxc13b* (Thummel et al., 2007), or *dlx* genes (Schebesta et al., 2006; Yoshinari et al., 2009) are potential genes to be mediating up-stream positional memory.

As stated above, this Fgfr1-dependent mechanism also controls adult tissue maintenance (Wills et al., 2008). In another interesting experiment, Poss' group inactivated Fgfr1 in adult tissues for 2 months. Following this inactivation, the distal fin gradually disappeared (Wills et al., 2008). This phenotype was also observed after *fgf20a* and downstream *mps1* inactivation (Wills et al., 2008). Thus, Mps1-dependent tissue maintenance is somehow quantitatively related to the proximodistal axis. These results initially support a genetic commonality between fin regeneration and tissues homeostasis (see Wills et al., 2008).

A secondary proximodistal patterning/size regulation is related to ray joint positioning. Fin rays of *cx43* mutant fishes show neighbor joints closer than wild type fins (Iovine and Johnson, 2000), whereas *alf*^*ty86d*^ fin rays show neighbor joints separated by a much larger, variable distance (Fig. 7G; Murciano et al., 2007; Sims et al., 2009). This evidence supports the proposal of a genetic control of joint positioning (JP, Borday et al., 2001; Géraudie and Birraux, 2003). In addition, different inter-joint patterns may be observed in neighbor *alf*^*ty86d*^ rays of equal size (Murciano et al., 2007). This final observation suggests that the genetic controls for joint positioning and proximodistal size are different at some point.

In the regeneration process, *cx43* (Fig. 7H; Sims et al., 2009) is expressed in distal/intermediate blastema positions. Morpholino knock-down of *cx43* in *alf*^*ty86d*^ mutant background shows epistatic *cx43* phenotype of closer joints during fin regeneration. Thus, *cx43* gene can be proposed to act downstream in *alf*^*ty86d*^ mutation. Moreover, distal/intermediate *cx43* expression is also expanded along the proximodistal axis of *alf*^*ty86d*^ fin blastema (Sims et al., 2009). This suggests that the distal/intermediate *cx43* expression might be involved in joint positioning (Sims et al., 2009; Fig. 7H,I).

In brief, the establishment of the proximodistal axis during the early patterning of the pectoral fin and the late morphogenesis of the tail fin are currently being studied. An RA and *fgf*-dependent patterning occurs in the pectoral fin bud. The proximodistal patterning is conserved in fishes and tetrapods. Moreover, a common *fgf*-signalling, *lof*^*dt2*^, or *cx43* control regulates late fin development, adulthood, and regeneration at the DGM. Such control regulates gene expression, fin morphogenesis, joint positioning, and/ or growth rate. These genes may act downstream of a quantitative pre-specification, which is proposed to be distributed in a proximodistal gradient and dependent on intertissue interactions at a distal organizer.

### Regulatory Interactions Along the Anteroposterior Axis

During late embryogenesis, the anteroposterior pattern of the tail fin has been shown to be regulated by the Bmp/Tolloid/Chordin pathway (Fisher and Halpern, 1999; see Connors et al., 1999) and a Hedgehog signal. Such a signal is different from Shh and released by the ACFP organizer (Hadzhiev et al., 2007). Additionally, pattern and size along the anteroposterior axis of paired fins have been shown to be established by both a *shh*-independent (e.g., Neumann et al., 1999; Yelon et al., 2000; Hoffman et al., 2002, Gibert et al., 2006) and a *shh*-dependent (Neumann et al., 1999; Hoffman et al., 2002) regulation by the ZPA (see Mercader, 2007). A “pre-pattern” is established by *hand2* gene, which is transcriptionally regulated by retinoic acid (Yelon et al., 2000). *hand2* gene regulates the position of Shh in a zone of polarizing activity (Fig. 1B, D). From the ZPA, Shh may diffuse into anterior mesenchyme regions. As suggested by abnormal expression under mutant backgrounds, the diffusion of RA and Shh generates collinear expressions of *hoxd11*, *hoxd12, hoxd13*, and *hoxc6* genes along the anteroposterior axis (Neumann et al., 1999; Yelon et al., 2000; Gibert et al., 2006; Sakamoto et al., 2009). Alterations in the ZPA also lead to anteroposterior defects in the skeleton (see Neumann et al., 1999; Grandel et al., 2000, 2002; Gibert et al., 2006; Sakamoto et al., 2009). This evidence suggests a hierarchy of genes controlling patterning and size along the anteroposterior axis of the fin bud.

Most gene mutations described above show both anteroposterior and proximodistal endoskeletal phenotypes. Interactions between the ZPA and the AER have been studied to be mediated by both Shh and Fgf4 signals (Grandel et al., 2000; Lee and Roy, 2006; Nomura et al., 2006; Prykhozhij and Neumann, 2008). These interactions might explain the observed complex phenotypes. The comparison between the genes regulating these two axes during fin (Fig. 5, see above) and tetrapod limb (see Saunders, 2002; Fernández-Terán and Ros, 2008) bud formation suggests evolutionary conservation (Mercader, 2007).

During the late development and regeneration of the tail fin, patterning and size control along the anteroposterior axis have also been disclosed. The tail fin of wild type zebrafish is anteroposteriorly symmetrical showing two lobes of similar size (Fig. 1G). Moreover, *eve1* and *evx2* genes are expressed in low levels in medial ray blastemas and in higher levels in large ray blastemas (see above; Brulfert et al., 1998). Furthermore, tail fin of *lof*^*dt2*^ mutant fish is anteroposteriorly asymmetrical showing a much larger anterior lobe (Fig. 7F; Géraudie et al., 1995). Nonetheless, this control of anteroposterior patterning and size is unable to promote fin regeneration along this axis (Morgan, 1902; Nabrit, 1929).

Several observations further support local interactions controlling growth rate along the anteroposterior axis. After oblique cuts (Morgan, 1902; Nabrit, 1929) or ray grafting (Eibner et al., 2008), ray blastema have been shown to activate growth in neighboring ones. This evidence suggests that local interactions control growth rate along the anteroposterior axis during fin regeneration. Finally, *sdf1/cxcr4a* genetic pathway has been proposed to be involved in cell division control and ray-interray interactions (Dufourcq and Vriz, 2006). Genetic pathways similar to *sdf1/cxcr4a* may initially provide an explanation to classical Morgan's experiments (1902).

**Fig. 8:**
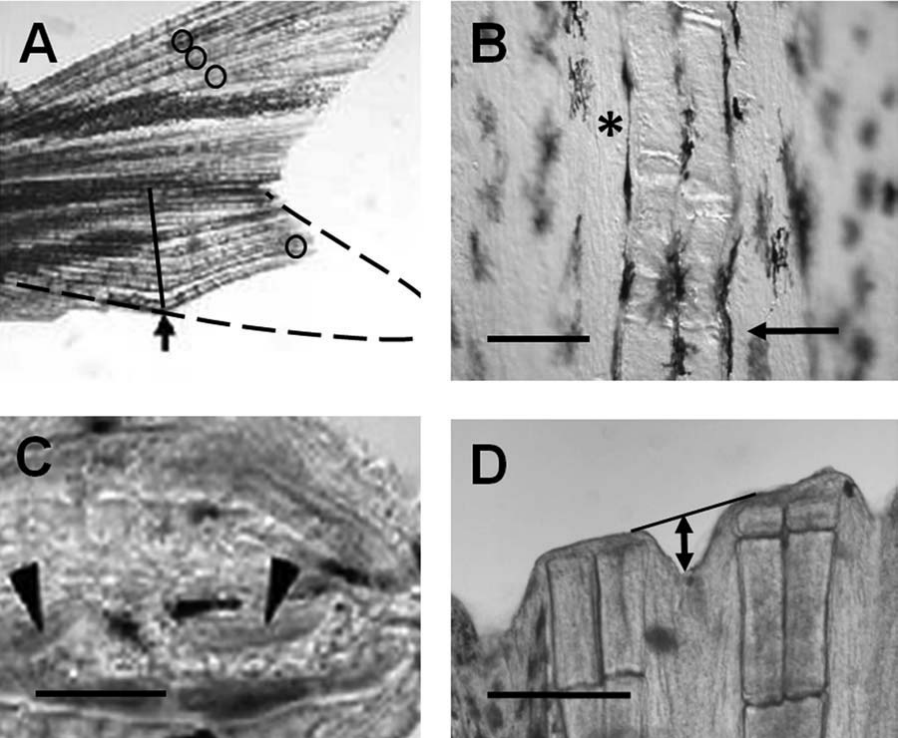
Phenotypes of pattern and differentiation gene perturbations. **A**: Heat-shocked regenerate of *hsp70:dn:fgfr1* tail fin. Reproduced from Lee et al. (2005) with permission of the publisher. The line and the arrow show where the cut took place. The discontinuous curved line shows the expected size of the outgrowing lobe. The circles show ray branching. **B**: Fusion phenotype (asterisk) obtained by injection of 0.2–0.6 nl of 100 ng/μl *shh* expression-plasmid in a branching ray blastema (according to Quint et al., 2002). Arrow shows cut plane. **C**: Cross-section of a *simplet* morphant ray. Arrowheads show ectopic lepidotrichia. Reproduced from Kizil et al. (2009) with permission of the publisher. **D**: Serrate phenotype (double arrow) obtained after 12-hr treatment with 5 μM Alk4/5/7-inhibitor SB431542 during wound healing (according to Jazwinska et al., 2007). Scale bar = 100 (C), 125 (B), and 250 (D) μm.

Some evidence further suggests that local interactions also regulate dermoskeleton patterning and size by cross-regulation of anteroposterior and proximodistal axes. Experimentally isolated rays do not branch and neighboring interrays are not formed (Murciano et al., 2002). Besides, non-branching lateralmost rays (R1, with one neighboring interray) may branch when experimentally regenerating with two neighboring interrays (see schematic details in Fig. 4A,B; Murciano et al., 2002). This evidence suggests local interactions controlling morphogenesis (Murciano et al., 2002). During non-experimental tail fin regeneration, a fan-like phenotype of distal expansion is generated. Under some experimental conditions, this fin expansion collapses showing convergence of the outgrowing rays. According to published phenotypes by Poss' group, Fgfr1 inactivation during fin regeneration may also lead to this “distal expansion collapse” (e.g., Fig. 8A reproduced from fig. 8B in Lee et al., 2005). This phenotype shows ray bending, reduction in ray and inter-joint length and ray/interray width, and distal positioning of ray branching. A somewhat similar phenotype is also observed in the fin regeneration process after exogenous administration of retinoic acid (White et al., 1994). This experiment leads to interray size reduction (Géraudie et al., 1993; White et al., 1994), apoptosis induction (Géraudie and Ferretti, 1997), and expression of ray-specific genes in distal interrays (Brulfert et al., 1998). However, *rar*γ and *fgfr1* are expressed in each ray blastema, not in the interrays (White et al., 1994; Poss et al., 2000). The potential lateral interactions might be mediated by diffusible signals such as Retinoic acid or Fgfr1. This might control distal widening, ray branching, and interray outgrowth by signaling neighboring tissues. This evidence suggests that proximodistal genes also control ray to interray morphogenesis regulating anteroposterior patterning and size.

Enough information supports a genetic control of proximodistal and anteroposterior fin axes. However, little evidence supports such a control along a third axis, the contralateral one, during these processes.

### Regulatory Interactions Along the Contralateral Axis

Symmetry between apposed hemirays is a general feature of all fins in *Danio rerio*. However, dorsal and ventral hemirays of the pectoral fin of zebrafish are different in thickness (Grandel and Schulte-Merker, 1998). By in situ hybridization, orthologous genes to those controlling dorsoventral axis during tetrapod limb bud formation (see Fernández-Terán and Ros, 2008) have also been involved in this process (Hatta et al., 1991; Ekker et al., 1992; Grandel et al., 2000; Norton et al., 2005; Mercader, 2007). Depending on signaling from the AER (Fig. 5; Grandel et al., 2000; Norton et al., 2005), *engrailed1* (*eng1a*) and *wnt7a* are, respectively, expressed in the non-AER ventral and dorsal ectoderm (Fig. 5; Hatta et al., 1991; Ekker et al., 1992). This molecular regulation is similar to that observed in tetrapod limb buds (see Fernández-Terán and Ros, 2008). However, no gene function has been studied on this interesting problem.

During late fin development and regeneration, some further evidence also supports a genetic control of this contralateral pattern. Contralateral developing and regenerating hemirays show both registered joint positions and symmetrical ray branching and length (Murciano et al., 2007). After ray transplantation, this symmetric pattern also occurs in regenerates from recombinant rays irrespective of graft origin (Fig. 4C–D; Murciano et al., 2007). Furthermore, *another long fin* (*alf*^*ty86d*^) mutation also modifies registered joint formation during development and regeneration (Fig. 7G, Murciano et al., 2007). This experimental and genetic evidence suggests that contralateral interactions may communally regulate ray pattern and size during both processes (Fig. 2B; Murciano et al., 2007).

All this evidence suggests an orthogonal genetic/intertissue control of three axes during fin morphogenesis (Fig. 2B). These interactions may communally regulate gene expression, pattern, and/or size during the three different morphogenetic processes. A genetic communality might also be proposed to occur between endoskeleton and dermoskeleton morphogenesis. A quantitative regulation of proximodistal morphogenesis, an Fgfs-dependent “pre-pattern,” or shh regulation by RA and Fgfs are conserved during both processes. In pectoral fin and tetrapod limb buds, this signaling has also been shown to be conserved (see Mercader, 2007; Hu and He, 2008; Dubouc and Logan, 2009). This might lead to new interesting hypotheses of genetic communality of fin/limb size and pattern control among vertebrates (see Mercader et al., 2005, 2006; Iovine, 2007). In zebrafish, the genetic pathways acting downstream of this DGM 3D-orthogonal system locally control ray, interray, and joint differentiation.

### Genes Controlling Ray and Interray Differentiation

In this article, all reviewed data on ray and interray differentiation have been obtained from regeneration experiments (Fig. 8B–D). During this process, the differentiating scleroblasts and the neighboring epidermis have been proposed to cross-interact (Marí-Beffa et al., 1996; Laforest et al., 1998; Quint et al., 2002). *shh* (Laforest et al., 1998; Quint et al., 2002), *ihha* (Avaron et al., 2006), and down-stream genes, *ptc1* and *bmp2b*, are expressed in both scleroblasts and the neighboring epidermis (Laforest et al., 1998). It was important to demonstrate that the over-expression of *shh* in interray blastema promotes the ectopic formation of subepidermal hemirays, the *ray fusion* phenotype, by such injection (Fig. 8B; Quint et al., 2002). Also by plasmid injection, *chordin* repression of Bmp2b activity in ray blastema impedes scleroblast differentiation (Smith et al., 2006). Furthermore, *shh* expression is restricted by *simplet* repressive function in the mesenchyme. After a morpholino knockdown of *simplet* gene, *shh* is also overexpressed in the internal blastemal cells, which are transformed into scleroblasts (Fig. 8C; Kizil et al., 2009). In principle, Shh release from epidermis to mesenchyme may organize Bmp2a induction of the scleroblast fate (Fig. 6; Quint et al., 2002). This signaling hypothesis is compatible with the observed absence of cell lineage restrictions (Poleo et al., 2001; Murciano et al., 2002, 2007) and similar to compartment patterning in the fruitfly (e.g., Marí-Beffa, 2005, and references within).

A genetic/pharmacological report also supports the notion of an independent genetic control of interray differentiation (Jaźwińska et al., 2007). ActβA ligand and Alk4 receptor are expressed in the distal interray during blastema formation. Experimental down-regulation of this pathway prevents interray differentiation, the *serrate* phenotype (Jaźwińska et al., 2007; Fig. 8D). According to this evidence, Hedgehog and ActβA-signaling pathways may independently control ray and interray differentiation (Fig. 6).

Retinoic acid and Fgfr1/Ras signaling pathways occur in the distal ray blastema (Figs. 6, 7E,H; White et al., 1994; Lee et al., 2009). These pathways have been proposed to restrict *shh* expression to proximal domains (Fig. 6; Laforest et al., 1998; Lee et al., 2009). These proximodistal interactions may regulate the formation of the DGM “compartments” (e.g., Yoshinari et al., 2009). Moreover, pharmacological reduction of Shh pathway unexpectedly impedes ray branching (Quint et al., 2002). This suggests complex regulatory interactions controlling ray/interray differentiation. *rar-γ, fgfr1*, but also *shh* or *actβ-A*, might control ray/interray morphogenesis regulating distal widening and lateral interactions. Potential activators/inhibitors from neighboring regions, such as Shh, might cross-interact at the ray-interray boundary organizer (see Murciano et al., personal communication). Distal widening, controlled by RA or *fgfr1*, might lead medial ray or interray regions to be below threshold concentration of diffusing signals mediating lateral activation. Thus, primary distal widening may secondarily lead to ray branching or interray formation by regulation of ray/interray differentiation genes (Murciano et al., 2002; personal communication).

This analysis aims to explain the ordered patterning of several gene expression domains along the proximodistal and anteroposterior axes of the blastema (Fig. 7E,H; e.g., Yoshinari et al., 2009; Brown et al., 2009). However, other expression domains have already been correlated with a last DGM function, the joint differentiation control.

### Genes Controlling Joint Differentiation

During zebrafish fin development and regeneration, ray joint differentiation occurs at distal regions. During both processes, further outgrowth does not affect the position and distance between already-formed joints (Fig. 1G,H; e.g., Géraudie et al., 1995).

During development, *evx1* (Borday et al., 2001), *hoxa13b* (Géraudie and Birraux, 2003), and *cx43* (Figs. 6, 7E,H; Sims et al., 2009) are expressed in the proximal blastema. This expression domain is named “joint field” and precedes the prospective position of ray joints (Sims et al., 2009). Beside these issues, no clear evidence supports any specific genetic control of joint differentiation in the “joint field” (Figs. 6, 7E,H,I; Sims et al., 2009). Interestingly enough, the above-mentioned joint-positioning regulator acting downstream the distal domain and upstream the “joint field” is still elusive.

Beside these genetic/intertissue controls at the DGM, several issues support further genetic control in the proximal growing domain.

## THE PROXIMAL GROWING DOMAIN

During fin development and regeneration, proximal ray and interray widening and thickening occur in all proximodistal positions away from the DGM. This process increases ray/interray width as body size augments (Fig. 1F–H). This also shows joint positioning maintenance by absence of ECM deposition in the joints (see Murciano et al., 2007).

**Fig. 9:**
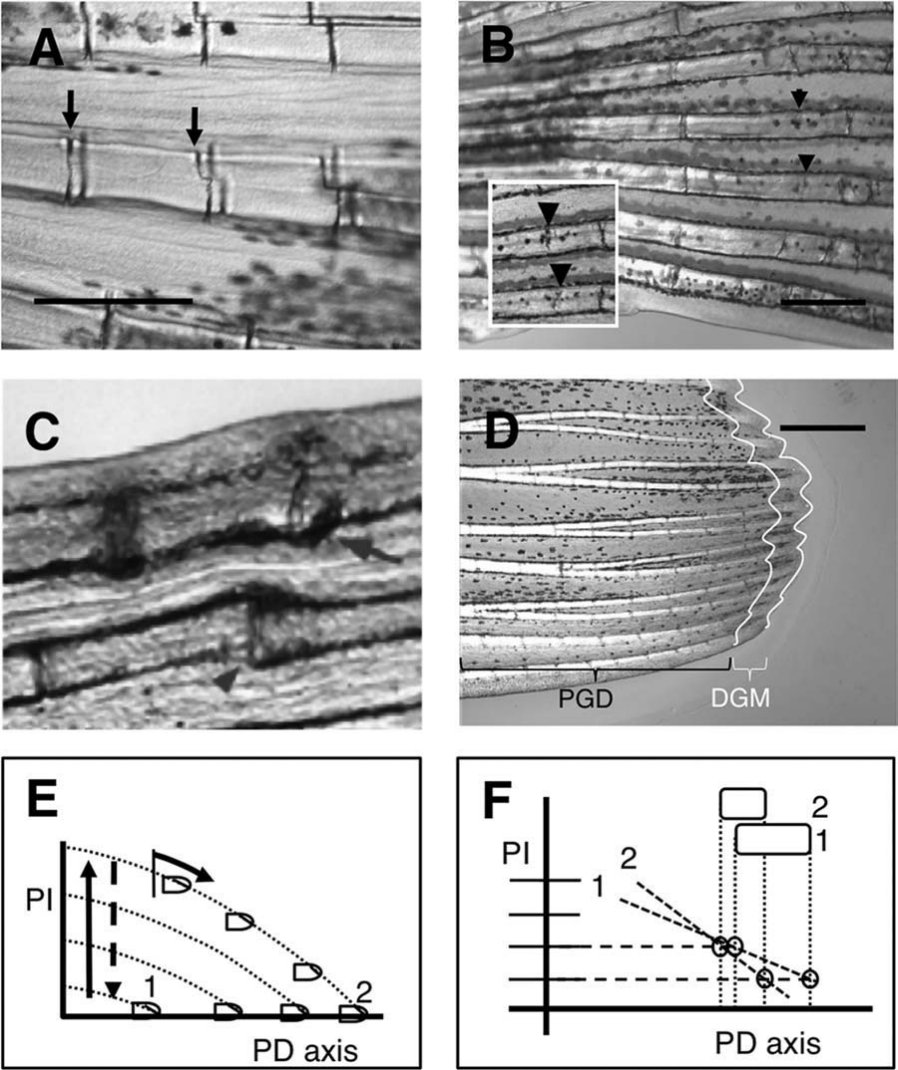
Positional model and experiments showing PGD. **A**: Step-like joints (arrows) in zebrafish tail fin. **B**: Proximoanterior regions of an *alf^ty86d^* tail fin regenerated for 22 days post-amputation (dpa). Inset shows the same joints regenerated for 14 dpa. Arrowheads indicate joint erasing (according to Murciano et al., 2007). **C**: Heat shock pulses in an adult tail fin of *hsp70:dn:fgfr1* fish for 2 months. Grey arrows show hypertrophic joints. Reproduced from Wills et al. (2008) with permission of the publisher. **D**: Growing domains in the wild type tail fin. DGM and PGD are as in text. **E**: Spatial positional model. Dotted curved lines are PI gradients. Up-pointing arrow shows PI increase during development. After cut (small vertical line), PI gradient regenerates (curved arrow). Bullet profiles are regenerating (top) or developing (bottom) DGM. 1 and 2 are proximodistal positions in F. Discontinuous arrow is experimental reduction of PI transduction (Wills et al., 2008). **F**: PI control of joint differentiation. Discontinuous oblique lines, PI slope at DGM; discontinuous horizontal lines, PI activity up-regulating periodic scleroblast repression by JP (circles). 1 and 2 are as in E. Rectangles show ray segment size generated at previous positions. PD is proximodistal. Scale bar = 300 (A) and 500 μm (B, D). Original figure (C) does not show bars.

Wild type rays in adult zebrafish fins may show stepped joints (Fig. 9A). These rays may form joints at different proximodistal levels after branching (see Fig. 9A). Ray/interray widening may fuse sister branches into a single ray, which now shows the step-like morphology (Fig. 9A). This process may gradually “distalize” ray branching-position as the body grows. A genetic mechanism may control the ray/interray width/thickness and joint maintenance in this proximal growing domain.

### Regulatory Control of Ray/Interray Width/Thickness and Joint Maintenance

Two genetic reports have dealt with proximal joint maintenance (Murciano et al., 2007; Wills et al., 2008). In the early development of fin dermoskeleton, *evx1* expression is conserved in proximal joints away from the DGM (Borday et al., 2001). This gene expression disappears in developed fins. A distal pattern of joints is transiently formed during tail fin regeneration of *alf*^*ty86d*^ fishes. Once the fin has regenerated, a proximal lepidotrichium-extracellular matrix deposition slightly widens the rays (Murciano et al., 2007). This process also erases some of these transient joints into the final pattern (Murciano et al., 2007; Fig. 9B). Furthermore, *hsp70:dn-fgfr1* over-expression during adulthood leads to a severe fin atrophy, which includes hypertrophic joint pathologies of scleroblast expansion (Fig. 9C; Wills et al., 2008). As *fgfr1* in situ hybridization does not stain proximal fin regions (Poss et al., 2000; Smith et al., 2008), this experiment suggests a remnant Fgfr1 function in the PGD. According to these data, *fgfr1* and *evx1* are transcriptional silenced at proximal positions. Moreover, the remnant Fgfr1 function might proximally repress joint positioning regulator. This evidence supports the notion of a genetic control of lepidotrichium extracellular matrix-deposition by proximal scleroblasts. This joint maintenance and gene transcription regulation could be part of a ray width/thickness control operating away from the DGM. Preliminary experiments suggest that proximal ray/interray width also depend on ray-interray boundary interactions (Murciano et al., personal communication).

A previous article, also proposed that histogenesis during *Salaria pavo* fin regeneration occurs by a similar process (Misof and Wagner, 1992). The proposed process has two steps: initial differentiation, which is similar to the distal patterning we have studied, and interactive structural maintenance, which is similar to the PGD activity (Misof and Wagner, 1992; Fig. 9D). In any case, a correct plain morphology is necessary for a well-adapted functional fin (Alben et al., 2007). The common Fgfr1 regulation at both the DGM and the PGD may co-ordinate growth in all fin positions. Besides the distal organizer, the proposed *pinnamere* and the ray-interray boundary organizer could also be local growth controlling units at both DGM and PGD (Murciano et al., personal communication). In order to explain this coordinated growth, we propose a positional model.

## POSITIONAL MODEL

In our model, we assume the existence of a pre-specification that controls position-dependent growth and differentiation, the “positional identity” gradient. This pre-specification gradient is transduced by Fgfr1 and RA at each ray DGM. Fgfr1 or RA modifications show mutant phenotypes along the proximodistal and anteroposterior axes. This evidence suggests that the pre-specification gradient might regulate gene expression, growth rate, and size (Fig. 7I; Lee et al., 2005; see Géraudie and Ferretti, 1997; Wills et al., 2008) along both axes.

In a feasible model, several processes regulate the “positional identity” gradient.

1. The “positional identity” gradient increases with body size (Fig. 9E). This increase is homogeneous in all proximodistal axis positions.
2. During gradient increase, a new distal position of the gradient might be generated by two different processes: “distalization” and relative reduction of the “positional identity” activity in newly generated distal positions.
3. The gradient activity may be directly proportional to several gene expressions and a steady state balance between cell division and apoptosis. A higher gradient would result in more cells in the steady state, and a lower gradient would lead to less final cells. This controlled balance may occur along the anteroposterior axis in the PGD (dependent on the ray/interray organizer) and along both axes at the DGM (also dependent on the distal organizer). When the gradient increases, both proximal widening and distal outgrowth may be caused by proportional regulation of the steady state balance. We suggest that fin length is acquired when the gradient regulates a null distal balance between cell division and apoptosis.
4. A joint positioning regulator may generate a scleroblasts repression in the joints by periodic activation in varying position identity activities (Figs. 7I, 9F). In order to generate a fixed periodicity, a constant JP activity may occur at maximal proximalmost PI activities. Variations in the distal reduction of gradient activity in the newly formed tissues may influence joint formation. If distal reduction generates a smooth slope, the neighboring inter-joint distance would be larger (1 in Fig. 9F). On the other hand, if distal reduction generates a sharp slope, the distance between neighbor joints would be shorter (2 in Fig. 9F). In this parabolic model, our potential joint positioning regulator may still be active in the PGD during joint maintenance.

We provide here the above-mentioned experimental evidence supporting this model.

1. During fin regeneration, *fgfr1*-downstream gene expression suggests a proximodistal gradient (Lee et al., 2005). Moreover, a genetic mechanism have been shown to control a balance between body and fin size have been shown (Iovine and Johnson, 2000). *lof*^*dt2*^ fins show an over-expression-dependent increase in ray length but wild type joint-positioning (see Géraudie et al., 1993; Iovine and Johnson, 2000; Green et al., 2009). In this mutant, a homogeneous increase in the gradient may occur maintaining the gradient slope in each position. This may lead to a miss-regulation of the cell division/apoptosis balance but not of the joint positioning regulator. Under our model, *lof* gene may regulate/transduce gradient increase with body size.
2. During fin regeneration, the pharmacological reduction of Shh pathway activity suggests the existence of dermoskeleton “distalization” (see Quint et al., 2002). Under exogenous administration of Shh pathway inhibitor, fin regenerates show normal morphology without distal regions, a “non-distalized” fin (see Quint et al., 2002). However, the relationship between Shh pathway and “distalization” is still elusive.
  *alf, cx43*, or *fgfr1* gene modifications show aberrations in both ray length and joint positioning (see Iovine and Johnson, 2000; Lee et al., 2005; Murciano et al., 2007; Sims et al., 2009). This may be explained by abnormal transduction of the distal reduction of the gradient at new positions. Changes in the gradient slope might be equally transduced in the miss-regulation of both the cell division/apoptosis balance and the joint positioning periodic regulation (e.g., *cx43* mutants). However, independent transduction of both processes may also occur in *alf*^*ty86d*^ mutant fins.
3. During early fin bud, ectodermal Fgf8 has been proposed to regulate both cell proliferation and apoptosis in underlying mesenchyme (Kawakami et al., 2003). During fin regeneration, Fgfr1 may regulate both epidermal apoptosis by retinoic acid synthesis (Géraudie and Ferretti, 1997; Mathew et al., 2009) and mesenchymal cell proliferation along the proximodistal axis by Mps1 (Nechiporuk et al., 2003; Fig. 7I). Dependent on distal epidermis-mesenchyme cross-interactions, Igf signaling has also been proposed to control fin outgrowth by regulation of both cell proliferation and apoptosis (Chablais and Jaźwińska, 2010). In our model, the gradient controls a cell division/apoptosis balance that regulates both growth rate and fin size at ray blastema. In addition, long-term over-expression of *hsp70:dn-fgfr1* adult fins may gradually decrease the transduction of the proximodistal gradient. The resulting reduction of adult fin size might be achieved by “proximalization” of the distal null balance (Fig. 9E; see Wills et al., 2008). In this condition, cell apoptosis may exceed cell division in distal positions (negative gradient values) leading to proximodistal size reduction.
  Furthermore, some experimental data suggest that the ray/interray boundaries may control ray and interray pattern and width (see Murciano et al., 2002, and personal communication). Retinoic acid administration (White et al., 1994) and Fgfr1 activity reduction (Lee et al., 2005) also leads to ray and interray width modifications. Under our model, the activity of the ray/interray boundary organizer may be also regulated by “positional identity” gradient. This may control another cell division/apoptosis balance along the anteroposterior axis.
  Finally, distal gene expression is almost non-existent during development and very high, in some instances as a gradient, during regeneration (Fig. 9E; Akimenko et al., 1995; Lee et al., 2005). Our model integrates these results as a direct proportionality between positional gradient and gene expression.
4. Murciano et al. (2007) suggested that fin ray length and joint positioning are independently regulated at distal and proximal regions of the fin. The distal joint positioning regulator may independently transduce positional gradient by Cx43 into joint differentiation. This may be caused by transcription regulation of *cx43/evx1/hoxa13b* genes (Fig. 7I). Furthermore, proximal over-activation (via reduction of Fgfr1 repressor activity) or reduction (modified in *alf*^*ty86d*^) of the silenced joint positioning regulator may induce either joint hypertrophy or erasing. Inter-joint distance varies along the proximodistal axis and does not change with body size (see Hass, 1962; Murciano et al., 2007; Sims et al., 2009). In our parabolic model, proximodistal variations in the gradient slope and its transduction by joint positioning regulator at DGM and PGD would explain these results (Fig. 9E,F).

A previous model on this topic supported that inter-joint number and distance are independently regulated (Iovine and Johnson, 2000). In our model, cell division/apoptosis and joint positioning are considered independent in the control of these fin characters (Fig. 7I; see Murciano et al., 2007). But, our model also provides predictions that are easy to test. Distal gene expression at the DGM of young fish or mutant modifications of gradient-dependent gene expressions are predictions to be easily verified in the future.

Similar models have been proposed in the fruitfly. In this organism, organ size control has been proposed to depend on a memory process (Nellen et al., 1996), on Dpp absolute concentration (Rogulja and Irvine, 2005) or on inhibitor and activator signals (Nijhout, 2003; see also Serrano and O'Farrell, 1997). These similarities with our model prompt the need of new techniques and experimental approaches for a proper comparison.

In order to obtain a correct model of fin morphogenesis, several technical difficulties must be solved. During fin regeneration, many reductions of gene functions from different groups may lead to a complete growth arrest (Poss et al., 2000; Quint et al., 2002; Lee et al., 2005; Stoick-Cooper et al., 2007). Future genetic models might consider the possible feedback-interactions between genes in different expression domains (see Fig. 7H; Padhi et al., 2004; Yoshinari et al., 2009, Lee et al., 2009; Brown et al., 2009). Genetic studies might be combined with gene expression studies and heterotypic grafting (between different genetic strains) or any alternative mosaic analysis. This may provide a tool to unravel the molecular nature of the proposed interactive control of fin morphogenesis.

## CONCLUSIONS

Several studies on fin form generation have been reviewed in this report. The morphogenesis of the dermal skeleton of the zebrafish fin mostly depends on mechanisms acting at the distal margin. Experimental studies suggest that intertissue interactions also take place along three axes. A molecular description of these local interactions is in progress in which a specific hierarchy of genetic controls regulates fin patterning, size and differentiation. In these studies, regulatory mechanisms of signal transduction, gene/microRNA transcription, and ionic coupling by gap junctions have been disclosed. The mechanisms acting during late fin development, regeneration, and tissue renewal have also been shown to be partially similar. Some evidence supports the existence of a proximal growing domain that would co-ordinate the generation of a plain functional form. This controlling commonality provides a very important framework to understand fin morphogenesis in future studies.

## SHORT-TERM PERSPECTIVES

Some future perspectives can be imagined from the description above. A large number of additional developmental genes will be studied to verify gene function conservation during vertebrate fin/limb transition. However, this study will also allow the scientific community to complete a molecular model of fin morphogenesis. Throughout this report, several specific questions arose to complete a general glimpse of this process. The concrete hierarchy of genetic control of the three axes of the fin acting in late fin development, adulthood, and regeneration must be analyzed. The potential involvement of a proximal control of ray/interray width and thickness and transcriptional silencing of gene functions also needs to be solved. The molecular mechanism controlling inter-joint distance is still elusive. Finally, the study of the emerging genetic hierarchy and potential feedback interactions are also progress highways for further research on this interesting field of developmental biology.
